# Evaluation of short- and long-term mortality prediction in patients undergoing hip fracture surgery using a biomarker-based interpretable predictive modeling approach: a retrospective cohort analysis

**DOI:** 10.1186/s12911-026-03797-3

**Published:** 2026-09-23

**Authors:** Sait Fatih Öner, Sevim Şenol Karataş, Oğuz Kağan Bulut, Ferhat Karataş

**Affiliations:** 1 Department of Anesthesiology, University of Health Sciences, Elazig Fethi Sekin City Hospital, Elazig, Turkey; 2 Department of Computer Engineering, Fırat University, Elazig, Turkey

**Keywords:** Hip fracture surgery, Mortality, Biomarker-based prediction, Logistic regression, Predictive modeling

## Abstract

**Purpose:**

The aim of this study was to develop an interpretable predictive modeling approach to predict short-term (three-month) and long-term (one-year) mortality in patients undergoing hip fracture surgery, and to evaluate the prognostic value of routinely available clinical and laboratory variables, including age, sex, lactate-to-albumin ratio (LAR), red cell distribution width–standard deviation (RDW-SD), and neutrophil-to-lymphocyte ratio (NLR).

**Methods:**

This retrospective cohort study included 1,000 patients who underwent hip fracture surgery. Demographic characteristics and preoperative laboratory parameters (LAR, RDW-SD, and NLR) were recorded. Three-month and one-year mortality were defined as the primary outcomes. Independent predictors of mortality were assessed using a multivariable logistic regression model implemented within an interpretable predictive modeling framework. Model performance was evaluated using receiver operating characteristic (ROC) curves, area under the curve (AUC), precision–recall AUC (PR-AUC), and Brier score. Calibration was assessed using calibration plots, and optimal probability thresholds were determined using the Youden J index.

**Results:**

Among the included patients, 17.4% died within three months and 24.6% within one year following surgery. Non-survivors were older and had higher LAR, RDW-SD, and NLR values compared with survivors (*p* < 0.001). In multivariable analyses, advanced age and elevated NLR were identified as independent predictors of both short-term and long-term mortality (*p* < 0.001). LAR was independently associated with one-year mortality (odds ratio: 1.035; 95% confidence interval: 1.008–1.063; *p* = 0.010). The model demonstrated acceptable discriminative performance, with an AUC of 0.807 for three-month mortality and 0.810 for one-year mortality. At the optimal thresholds, sensitivity and specificity were 80% and 73% for three-month mortality, and 77% and 71% for one-year mortality, respectively.

**Conclusion:**

The biomarker-based interpretable predictive modeling approach developed in this study demonstrated acceptable discriminative performance for estimating short- and long-term mortality after hip fracture surgery. Advanced age, elevated NLR, and increased LAR emerged as important predictors. As these variables are routinely available in clinical practice, the proposed framework may support perioperative risk awareness and assist clinical evaluation when interpreted together with comprehensive clinical assessment.

**Clinical trial number:**

Not applicable.

## Introduction

Hip fractures are a common health problem, particularly in the older population, and are frequently associated with substantial morbidity and mortality, often necessitating surgical intervention. In elderly patients, one-year mortality rates following hip fracture surgery have been reported to reach up to 20–30% [1].

Accurate prediction of mortality risk in patients undergoing hip fracture surgery is therefore of considerable importance for perioperative management and treatment planning. Conventional clinical risk scores provide only moderate predictive accuracy and may fail to adequately capture individual variability in risk [2]. In recent years, predictive modeling approaches, including regression-based and machine learning methods, have been increasingly applied to clinical risk prediction, particularly in settings involving complex, multivariable data structures. This trend has been driven in part by the ability of these methods to account for nonlinear and interacting relationships among clinical variables [3].

Several studies have reported that machine learning–based models achieve higher discriminative performance in predicting postoperative mortality compared with traditional risk scoring systems [4]. For instance, random forest algorithms have been successfully applied to estimate one-year mortality risk following hip fracture surgery [5]. Beyond mortality prediction, machine learning approaches have also been used to estimate long-term functional outcomes in this patient population [6]. Moreover, large-scale and multicenter studies comparing different machine learning algorithms have suggested that certain models may enhance clinical risk stratification [7].

Despite these advances, the clinical applicability of many machine learning models remains limited by their complex and opaque structures, which can hinder interpretability in clinical decision-making. When evaluating high-stakes outcomes such as mortality, model performance should not be judged solely on discriminative ability, but also on transparency and clinical interpretability. Accordingly, there has been growing interest in predictive models whose decision-making processes can be more readily examined and understood within a clinical context.

In this context, developing an interpretable predictive model to predict one-year mortality in patients undergoing hip fracture surgery may offer meaningful support to existing clinical practice. The present study was conducted with this objective in mind.

## Materials and methods

The study was approved by the Ethics Committee of Elazığ Fethi Sekin City Hospital (Approval No: 2025/20–22; Date: December 4, 2025). Due to the retrospective nature of the study, the requirement for informed consent was waived. All procedures were conducted in accordance with the principles of the Declaration of Helsinki.

This retrospective, observational cohort study was conducted at Elazığ Fethi Sekin City Hospital and included patients aged 18 years and older who underwent surgical treatment for hip fracture between January 1, 2019, and January 1, 2025. The diagnosis of hip fracture was confirmed using hospital electronic medical records based on ICD-10 codes S72.0 (fracture of neck of femur), S72.1 (pertrochanteric fracture), and S72.2 (subtrochanteric fracture).

**Fig. 1:**
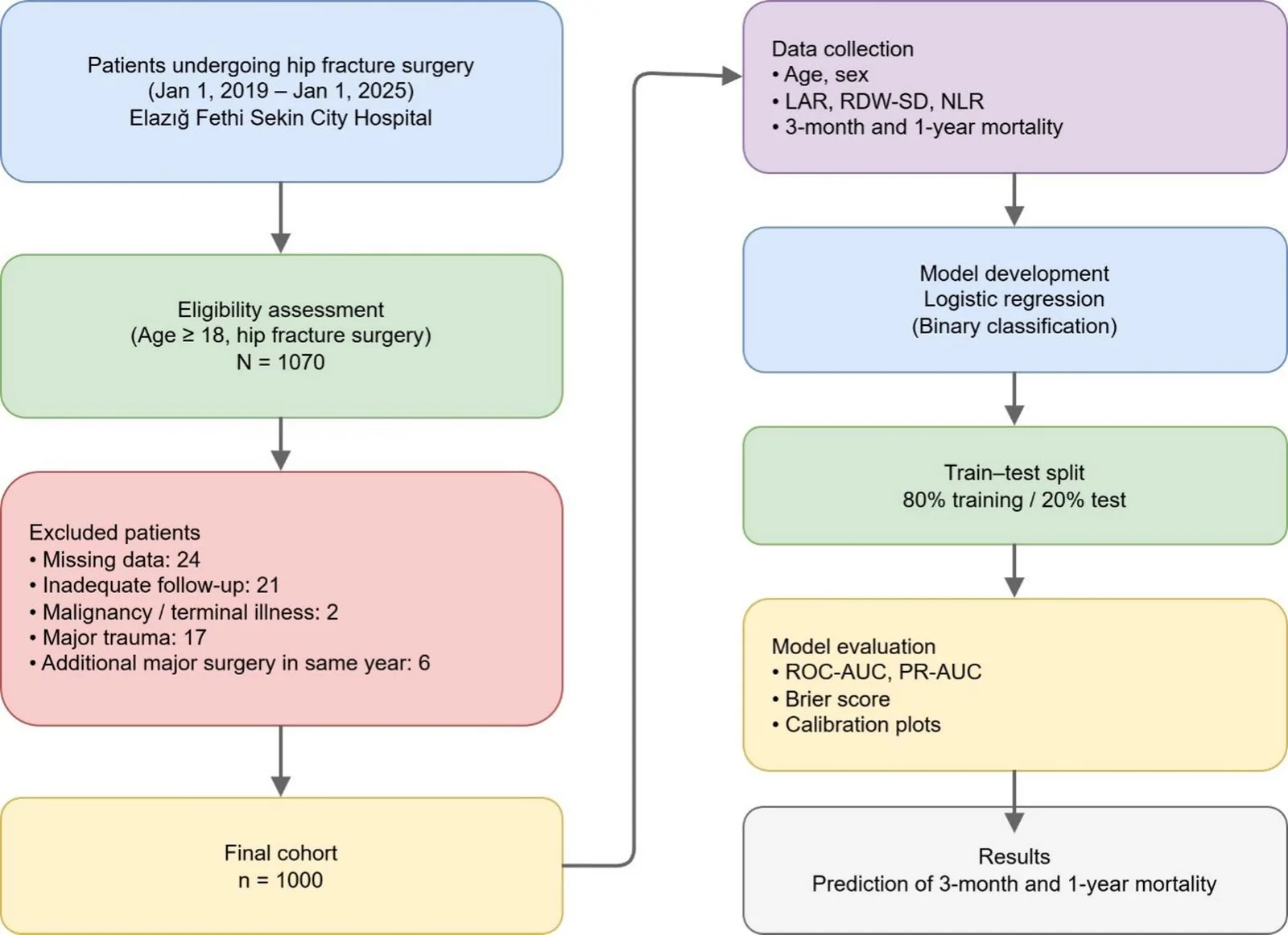
Study flowchart and model development workflow. This figure summarizes the patient selection process and analytical workflow. A total of 1,000 patients undergoing hip fracture surgery were included. Routinely available preoperative variables were used to develop an interpretable logistic regression–based predictive modeling framework for 3-month and 1-year mortality, with performance assessed using discrimination and calibration metrics

After applying the exclusion criteria—including incomplete laboratory data, insufficient follow-up duration, history of major trauma, presence of malignancy or terminal illness, and additional major surgical procedures within one year—a total of 1,000 patients constituted the final study cohort (Fig. 1). The proportion of missing data across study variables was below 2%, with missingness observed predominantly in laboratory parameters. Patients with missing laboratory or outcome data were excluded using a complete-case analysis approach. No imputation procedure was applied because the overall proportion of missing data was low and the analysis was restricted to objectively documented variables. Clinical and laboratory data were collected, after which an interpretable predictive model–based mortality prediction framework was developed and internally validated. One-year mortality status for all patients was verified using hospital records and the Central Civil Registration System (MERNİS).

Inclusion criteria were defined as age ≥ 18 years, having undergone surgery for hip fracture, availability of complete preoperative biochemical and complete blood count data, and the presence of at least one year of mortality follow-up. Exclusion criteria included incomplete clinical or laboratory data, age under 18 years, surgery for pathological fractures or malignancy-related fractures, and the occurrence of more than one major surgical intervention within the same year.

Demographic and clinical characteristics, preoperative biochemical parameters, complete blood count indices, derived inflammatory markers—including the neutrophil-to-lymphocyte ratio (NLR) and lactate-to-albumin ratio (LAR)—and outcome variables (three-month and one-year mortality) were recorded. Preoperative laboratory values were obtained from the first available blood samples collected at hospital admission before surgical intervention. Several clinically relevant variables, such as the American Society of Anesthesiologists (ASA) physical status score, Charlson Comorbidity Index, frailty scores, and time from admission to surgery, were not included in the model. This decision was primarily based on incomplete, heterogeneous, or non-standardized documentation of these variables in retrospective hospital records. To minimize information bias and enhance model robustness, only variables with high availability, objective measurement, and reliable documentation were incorporated. In addition, the study aimed to develop a parsimonious predictive model based on routinely available preoperative data.

All biochemical and hematological measurements were obtained as part of routine preoperative clinical evaluation and were analyzed in the hospital’s central accredited laboratory using standardized institutional measurement protocols and internal quality-control procedures. Because of the retrospective design, strict physiologic standardization of rapidly changing biomarkers such as lactate could not be fully ensured. To improve consistency, only preoperative measurements obtained before surgical intervention were included in the analysis. An interpretable predictive modeling approach was employed to estimate mortality risk in patients undergoing hip fracture surgery. Model input variables were selected based on clinical relevance and supporting evidence from the literature and included age, sex, lactate-to-albumin ratio (LAR), red cell distribution width–standard deviation (RDW-SD), and neutrophil-to-lymphocyte ratio (NLR). To facilitate clinical interpretability, the LAR variable was rescaled to represent increments of 0.01 due to its narrow numerical range.

Mortality prediction was formulated as a binary classification problem. Given the objective of developing a clinically interpretable and applicable predictive model, logistic regression was selected as the modeling approach. The model was evaluated within a supervised learning framework using separate training and independent test datasets, and performance was reported using metrics reflecting both discriminative ability and calibration. Accordingly, logistic regression was considered an interpretable predictive model within a supervised learning–based analytical framework.

Logistic regression was selected a priori as the primary modeling approach because it provides transparent coefficient estimates, directly interpretable odds ratios, and clinically meaningful probability outputs. Compared with more complex black-box algorithms, logistic regression allows clinicians to examine the direction and magnitude of association for each predictor, which is particularly important when modeling high-stakes outcomes such as postoperative mortality. Therefore, the present analysis was framed as an interpretable biomarker-based predictive modeling approach rather than as a complex machine-learning classifier.

All analyses were performed using Python (version 3.10). Data preprocessing, model training, and performance evaluation were conducted using the scikit-learn library, while coefficient estimates, odds ratios, and 95% confidence intervals were verified using the statsmodels package. Continuous variables were entered into the model using their original clinical scale to preserve the interpretability of odds ratios.

To reduce the risk of overfitting, the dataset was split into training and test sets while preserving the mortality event rate. Specifically, 80% of the data were allocated to the training set and 20% to the test set using stratified sampling. Model training was conducted exclusively on the training data, and performance was assessed on the independent test set. Separate models were developed for three-month and one-year mortality, each using the same training–test partitioning strategy.

Given the relatively large sample size and the objective of maintaining methodological transparency, a hold-out training–test validation strategy was preferred rather than repeated cross-validation. To further limit overfitting, only a restricted set of clinically meaningful and literature-supported variables was included in the models. Model performance was reported for both the training and independent test datasets. Calibration was evaluated using calibration plots and the Brier score. In addition, calibration slope and calibration-in-the-large (intercept) were calculated to provide a quantitative assessment of calibration.

Model performance was assessed using the area under the receiver operating characteristic curve (ROC-AUC), area under the precision–recall curve (PR-AUC), Brier score, sensitivity, specificity, accuracy, and F1-score [8]. The optimal classification threshold was determined using the Youden J index derived from the ROC curve [9]. Agreement between predicted probabilities and observed mortality rates was further examined using calibration plots, and overall calibration performance was quantified using the Brier score [10].

### Statistical analysis

The distribution of continuous variables was evaluated using the Kolmogorov–Smirnov and Shapiro–Wilk tests. Variables not following a normal distribution were presented as median with interquartile range (IQR). Comparisons between groups were performed using the Mann–Whitney U test for continuous variables and the chi-square test for categorical variables. Variables associated with mortality in univariable analyses and supported by clinical relevance and existing literature were included in multivariable models. A two-sided p value < 0.05 was considered statistically significant for all analyses.

## Results

A total of 1,000 patients were included in the study. During the three-month follow-up period, 826 patients survived and 174 patients died. At one-year follow-up, 754 patients were alive, while 246 patients were recorded as deceased.

Baseline clinical and laboratory characteristics of the study cohort are presented in Table 1. When patients were compared according to three-month mortality status, non-survivors were found to be significantly older than survivors (median 84.0 [77.0–89.0] vs. 76.0 [68.0–83.0] years; *p* < 0.001). Similarly, LAR, RDW-SD, and NLR values were significantly higher among patients who died within three months compared with those who survived (all *p* < 0.001).

**Table 1:** Baseline characteristics of the study population stratified by 3-month and 1-year mortality

Variable	Total cohort (*n* = 1000)	3-month survivors (*n* = 826)	3-month non-survivors (*n* = 174)	*p*	1-year survivors (*n* = 754)	1-year non-survivors (*n* = 246)	*p*
Age, years	79.0 [71.0–85.0]	76.0 [68.0–83.0]	84.0 [77.0–89.0]	< 0.001	75.0 [67.0–82.0]	83.0 [76.0–88.0]	< 0.001
LAR	0.04 [0.03–0.05]	0.03 [0.02–0.04]	0.06 [0.04–0.08]	< 0.001	0.03 [0.02–0.04]	0.05 [0.04–0.07]	< 0.001
RDW-SD	45.1 [42.4–48.1]	44.6 [42.0-47.3]	45.7 [43.3–49.4]	< 0.001	44.4 [41.9–47.2]	45.9 [43.4–49.6]	< 0.001
NLR	5.86 [3.24–10.4]	5.47 [3.10-10.14]	7.26 [3.85–11.7]	< 0.001	5.29 [3.01–9.56]	7.12 [3.96–11.6]	< 0.001
Female	631	536 (64.9%)	102 (58.8%)	0.084	514 (68.2%)	134 (54.4%)	< 0.001
Male	369	290 (35.1%)	72 (41.2%)	0.084	240 (31.8%)	112 (45.6%)	< 0.001

Continuous variables are presented as median [interquartile range]

Categorical variables are presented as n (%), with percentages calculated per column

P-values were calculated using the Mann–Whitney U test for continuous variables and the chi-square test for categorical variables

A comparable pattern was observed for one-year mortality. Patients who died within one year were older (83.0 [76.0–88.0] vs. 75.0 [67.0–82.0] years; *p* < 0.001) and had significantly higher LAR, RDW-SD, and NLR values (all *p* < 0.001). Although sex distribution did not differ significantly between survivors and non-survivors for three-month mortality (*p* = 0.084), male sex was more frequently observed in the non-survivor group in the one-year mortality analysis (*p* < 0.001) (Table 1).

The results of multivariable logistic regression analyses for predicting three-month and one-year mortality are summarized in Table 2. For three-month mortality, advanced age (odds ratio [OR]: 1.081; 95% confidence interval [CI]: 1.061–1.101; *p* < 0.001) and elevated NLR (OR: 1.373; 95% CI: 1.283–1.469; *p* < 0.001) were identified as independent predictors. LAR, RDW-SD, and sex did not reach statistical significance in this model.

**Table 2:** Multivariable logistic regression analysis for prediction of 3-month and 1-year mortality

Variable	OR (3-month)	95% CI	*p*	OR (1-year)	95% CI	*p*
Age (per 1-year increase)	**1.081**	1.061–1.101	< 0.001	**1.079**	1.061–1.098	< 0.001
Female vs. Male	1.298	0.939–1.793	0.114	1.144	0.837–1.564	0.398
LAR (per 0.01 increase)	1.023	0.996–1.052	0.099	**1.035**	1.008–1.063	0.010
RDW-SD (per 1-unit increase)	1.006	0.982–1.030	0.625	1.009	0.986–1.032	0.434
NLR (per 1-unit increase)	**1.373**	1.283–1.469	< 0.001	**1.420**	1.323–1.524	< 0.001

Odds ratios (ORs) are presented with 95% confidence intervals (CI)

LAR values were rescaled by a factor of 0.01 to improve interpretability of odds ratios

In the one-year mortality analysis, advanced age (OR: 1.079; 95% CI: 1.061–1.098; *p* < 0.001), elevated NLR (OR: 1.420; 95% CI: 1.323–1.524; *p* < 0.001), and increased LAR (per 0.01-unit increase; OR: 1.035; 95% CI: 1.008–1.063; *p* = 0.010) were identified as independent predictors. RDW-SD and sex were not significantly associated with one-year mortality (Table 2).

**Fig. 2:**
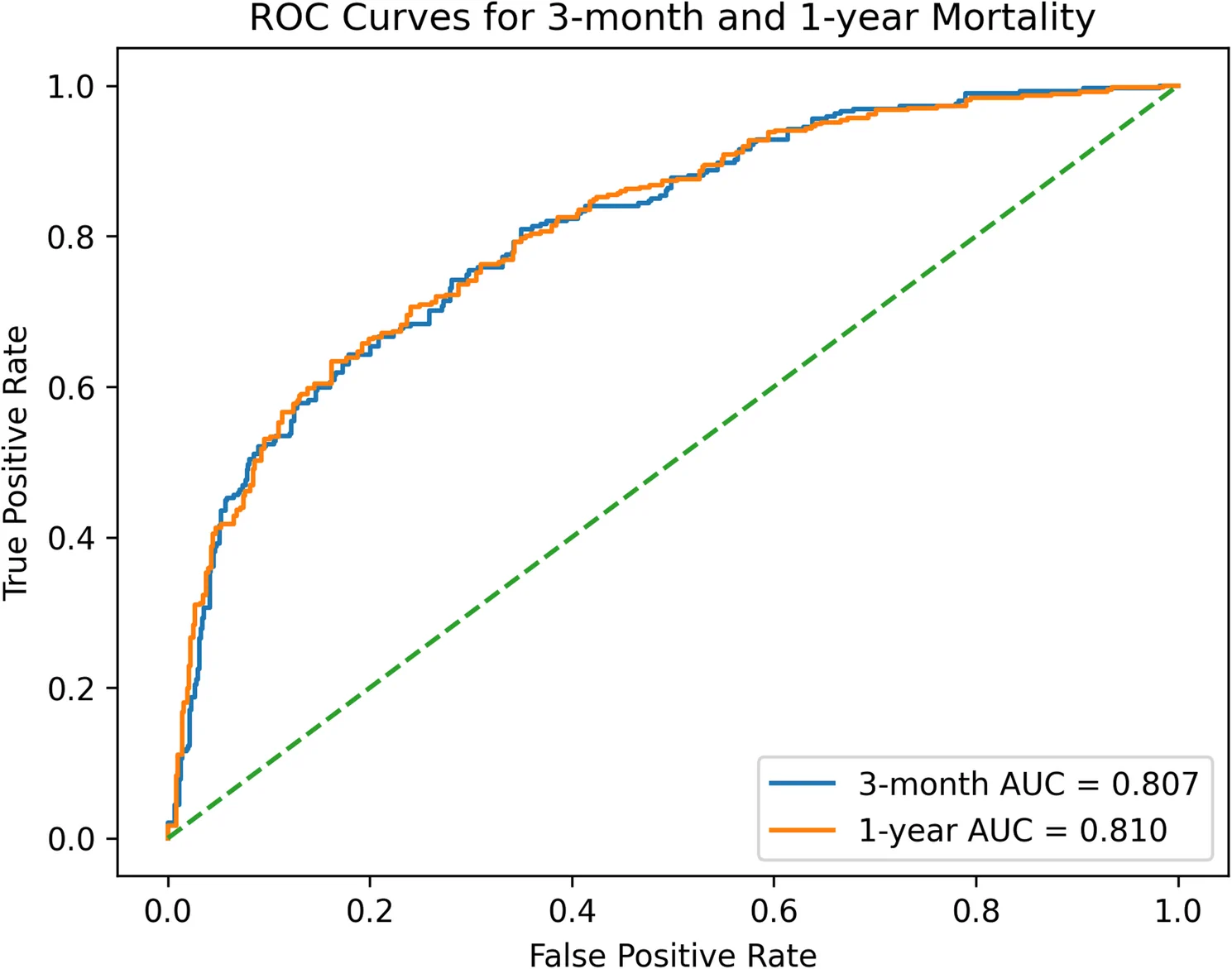
Receiver operating characteristic (ROC) curves of the multivariable logistic regression model for prediction of 3-month and 1-year mortality. The model included age, sex, LAR (per 0.01 increase), RDW-SD, and NLR. The area under the curve (AUC) was 0.807 for 3-month mortality and 0.810 for 1-year mortality

**Fig. 3:**
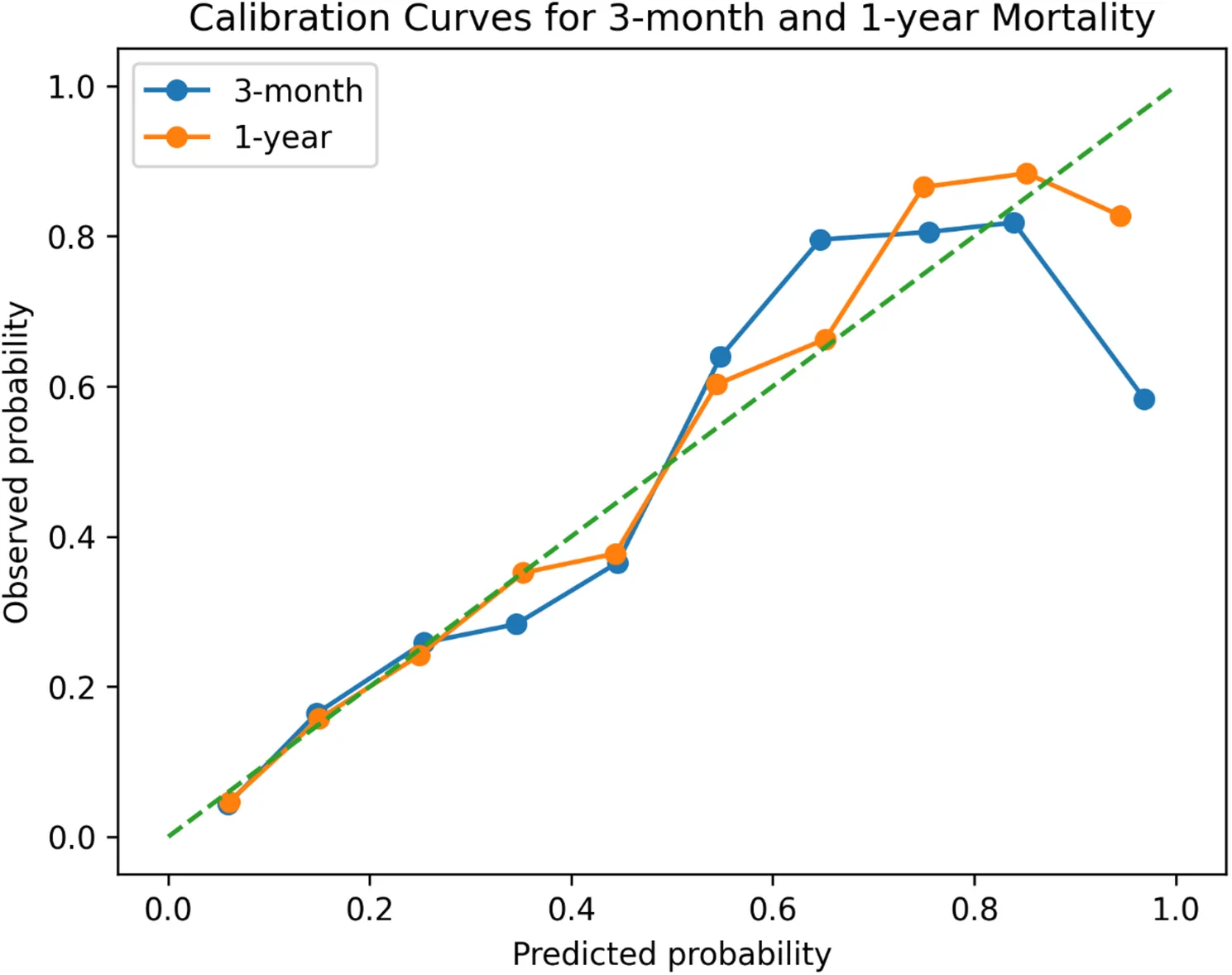
Calibration plots of the multivariable logistic regression model for 3-month and 1-year mortality. The model demonstrates good agreement between predicted and observed mortality risks across both short-term and long-term follow-up periods

The discriminative performance of the multivariable models was evaluated using receiver operating characteristic (ROC) curves (Fig. 2). The area under the ROC curve (AUC) was 0.807 for three-month mortality and 0.810 for one-year mortality, indicating acceptable discriminative performance for both time horizons. Model calibration is presented in Fig. 3, demonstrating good agreement between predicted risks and observed mortality rates for both short- and long-term outcomes.

In the test dataset, the calibration-in-the-large values were − 0.014 for three-month mortality and − 0.044 for one-year mortality, suggesting overall consistency between predicted probabilities and observed event rates. Calibration slope values were 0.785 and 0.892 for three-month and one-year mortality, respectively, indicating acceptable calibration performance across both models.

Detailed performance metrics of the models are provided in Table 3. For three-month mortality, the ROC-AUC was 0.807, PR-AUC was 0.651, and the Brier score was 0.168. Using the optimal probability threshold of 0.41, sensitivity was 80%, specificity was 73%, precision was 55%, and the F1-score was 65%. For one-year mortality, the ROC-AUC was 0.810, PR-AUC was 0.741, and the Brier score was 0.176. The optimal threshold was determined as 0.44, yielding a sensitivity of 77%, specificity of 71%, precision of 69%, and an F1-score of 73% (Table 3).

**Table 3:** Predictive performance of the multivariable logistic regression model for 3-month and 1-year mortality

Outcome	ROC-AUC	PR-AUC	Brier score	Sensitivity	Specificity	Precision	F1-score	Threshold
3-month mortality	**0.807**	**0.651**	0.168	0.80	0.73	0.55	0.65	0.41
1-year mortality	**0.810**	**0.741**	0.176	0.77	0.71	0.69	0.73	0.44

The optimal probability thresholds were determined using the Youden J index. AUC: area under the receiver operating characteristic curve

Classification performance based on the confusion matrix is shown in Table 4. For three-month mortality, 103 true negatives, 47 true positives, 38 false positives, and 12 false negatives were identified, corresponding to a false-negative rate of 20.3%. For one-year mortality, 101 true negatives, 59 true positives, 41 false positives, and 19 false negatives were observed, resulting in a false-negative rate of 24.4% (Table 4).

**Table 4:** Confusion matrix and classification performance of the multivariable logistic regression model for prediction of 3-month and 1-year mortality

Outcome	Threshold	TN	FP	FN	TP	Sensitivity	Specificity	False-negative rate
3-month mortality	0.41	103	38	12	47	0.80	0.73	20.3%
1-year mortality	0.44	101	41	19	59	0.76	0.71	24.4%

TN: true negative; FP: false positive; FN: false negative; TP: true positive

FNR: false-negative rate

The optimal probability thresholds were determined using the Youden J index

## Discussion

### Comparison with the literature

Although time-to-event modeling approaches such as Cox proportional hazards regression may provide additional insight into the temporal dynamics of mortality risk, the present study focused on predicting mortality at clinically meaningful fixed time horizons (three months and one year). This approach is frequently used in clinical prediction modeling, as it allows direct estimation of absolute risk probabilities at predefined time points, which may be more readily interpretable for perioperative risk stratification.

The discriminative performance achieved by the model developed in this study is generally consistent with findings reported in recent studies on mortality prediction in elderly patients undergoing hip fracture surgery [11, 12]. In the contemporary literature, machine learning–based models have typically reported ROC-AUC values in the range of 0.75–0.85 [13, 14]. In this context, the AUC values exceeding 0.80 for both short- and long-term outcomes in the present study suggest that the model’s performance aligns with previously published results. In addition, previously reported discriminative performance values for conventional clinical scoring systems such as the Nottingham Hip Fracture Score have generally ranged between approximately 0.70 and 0.78 in elderly hip fracture populations. Although a direct head-to-head comparison was not feasible in the present retrospective dataset, the observed AUC values of approximately 0.81 suggest that the proposed biomarker-based framework demonstrated comparable discriminatory performance within the limitations of the available variables.

The selection of three-month and one-year mortality endpoints was based on their established clinical relevance in hip fracture research. Three-month mortality is commonly considered an indicator of early postoperative vulnerability and perioperative physiologic stress, whereas one-year mortality reflects longer-term functional decline, frailty progression, and overall recovery trajectory following hip fracture surgery.

The identification of NLR as an independent predictor of mortality is in line with prior studies highlighting the role of the inflammatory response in postoperative survival following hip fracture surgery [15, 16]. Similarly, the association between LAR and long-term mortality is consistent with reports emphasizing the impact of metabolic stress and nutritional status on late outcomes [17]. In contrast, RDW-SD did not retain statistical significance in multivariable analyses, which may indicate that its prognostic contribution is attenuated when more dominant factors such as age and systemic inflammation are taken into account [18].

### Algorithmic risk and model overconfidence

In clinical predictive modeling, algorithmic overconfidence and inadequate calibration may increase the risk of erroneous clinical decisions when model outputs are interpreted without appropriate clinical context. The logistic regression–based approach adopted in this study offers a more predictable and controllable structure compared with complex black-box models. By providing probability-based outputs, the model facilitates risk assessment rather than binary decision-making, allowing clinicians to contextualize predictions within the broader clinical picture [19].

The observation that false-negative rates remained below 25% suggests a relatively low likelihood of failing to identify high-risk patients. Nevertheless, algorithmic outputs that are interpreted in isolation from clinical judgment may still pose safety concerns. Accordingly, the findings underscore that such models should not be used as stand-alone decision-making tools, but rather as supportive instruments that complement clinical evaluation and expertise [20].

The calibration slope values below 1 observed in this study (0.785 for three-month mortality and 0.892 for one-year mortality) may suggest a modest degree of model overfitting, particularly in higher-risk individuals. Such findings are commonly observed in internally validated prediction models and indicate that predicted probabilities may be slightly overestimated in some patient subgroups. Nevertheless, the overall calibration performance remained acceptable, as supported by the calibration plots and Brier scores.

### Clinical implications and anesthesiology-specific considerations

The findings of this study offer several implications relevant to anesthesiology practice. Preoperative risk stratification based on readily available parameters such as age, NLR, and LAR may enable earlier identification of patients at increased risk of mortality, thereby informing perioperative planning and anesthetic management [21, 22].

In particular, in older patients with a high burden of comorbidities, more vigilant hemodynamic management and structured postoperative monitoring protocols may facilitate earlier detection of complications and potentially improve outcomes [23]. In this regard, the proposed model may be viewed as a decision-support tool that enhances perioperative risk awareness rather than replacing clinical judgment.

### Strengths

One of the main strengths of this study is that the model relies exclusively on routinely available clinical and laboratory parameters, enabling broad applicability without additional costs or the need for advanced technological infrastructure. Furthermore, the reporting of both discriminative and calibration performance metrics enhances the transparency and credibility of the findings.

The deliberate use of a clinically interpretable modeling approach represents an additional strength. In the context of critical outcomes such as mortality, model explainability may play an important role in fostering clinician trust and facilitating integration into clinical workflows [24].

### Limitations and potential biases

Because several established clinical predictors of mortality after hip fracture surgery—such as ASA physical status, Charlson Comorbidity Index, frailty measures, perioperative timing variables, and functional status assessments—were not consistently available in the retrospective dataset, the present framework should be interpreted as a biomarker-based exploratory prediction tool rather than a comprehensive clinical risk model. The primary aim of the study was to evaluate the prognostic contribution of routinely available laboratory-derived biomarkers within an interpretable modeling framework.

Accordingly, the findings should be interpreted as supportive evidence regarding biomarker-associated mortality risk rather than as a stand-alone clinical decision-making system. Clinical interpretation should always be integrated with comprehensive perioperative assessment and physician judgment.

Several limitations of this study should be acknowledged. Because of the retrospective design, detailed information regarding short-term dietary intake, use of medications potentially influencing lactate or albumin metabolism, hydration status, and other transient physiologic factors was not consistently available for all patients. Therefore, residual confounding related to unmeasured metabolic, pharmacologic, or nutritional variables cannot be completely excluded. In addition, standardized documentation regarding palliative-care status, treatment-limitation decisions, or end-of-life care strategies was not consistently available in the retrospective records. Consequently, mortality outcomes were evaluated irrespective of differences in treatment intensity or goals of care. The retrospective and single-center design may introduce selection bias. Reliance on historical records may have precluded the inclusion of certain clinically relevant variables. In addition, the absence of external validation limits the generalizability of the findings. Although the separation of training and test datasets provides insight into internal consistency and performance stability, it does not ensure equivalent performance across different institutions or patient populations.

Another limitation of the present study is the absence of direct comparison with established hip fracture mortality prediction tools, such as the Nottingham Hip Fracture Score. Calculation of these established scores requires variables including ASA physical status, comorbidity indices, fracture type, and perioperative timing parameters, which were not consistently available in the retrospective dataset used in this study. Therefore, a head-to-head comparison with existing clinical risk scores was not feasible. The present study also did not include a non-operative comparison group. Therefore, the findings should not be interpreted as evidence regarding the survival benefit of surgical versus conservative management strategies. In current clinical practice, surgical treatment remains the standard-of-care approach for the majority of medically eligible hip fracture patients, and the present analysis was limited to mortality prediction within the surgically treated cohort.

Variations in patient characteristics, laboratory measurement practices, and perioperative care protocols may affect model transportability and external validity. Consequently, the reported performance metrics primarily reflect internal validity, and independent multicenter external validation is required before routine clinical implementation. In particular, external validation using independent multicenter datasets is essential to confirm the stability and generalizability of the proposed model. Therefore, the present findings should be interpreted as internally validated results that require further confirmation before routine clinical implementation. Moreover, the exclusion of frailty-related measures, which may substantially influence mortality risk, could result in different model performance under real-world conditions.

### Future directions

Future research should prioritize prospective, multicenter studies to externally validate the model and assess its generalizability. Integration into clinical decision-support systems and evaluation under real-time clinical conditions represent additional areas for further investigation [25].

## Conclusion

This study suggests that short- and long-term mortality following hip fracture surgery may be estimated using routinely available laboratory-derived biomarkers within an interpretable predictive modeling framework. Age, NLR, and LAR emerged as the most relevant predictors in the present cohort. Although the proposed biomarker-based approach demonstrated acceptable discriminatory performance, the findings should be interpreted within the limitations of the retrospective single-center design and the absence of several established clinical risk variables. Further prospective multicenter external validation studies are required before routine clinical implementation.

## Data Availability

The datasets used and/or analyzed during the current study are available from the corresponding author on reasonable request.
